# Comparing responsiveness of the EQ-5D-5L, EQ-5D-3L and EQ VAS in stroke patients

**DOI:** 10.1007/s11136-014-0873-7

**Published:** 2014-11-26

**Authors:** Dominik Golicki, Maciej Niewada, Anna Karlińska, Julia Buczek, Adam Kobayashi, M. F. Janssen, A. Simon Pickard

**Affiliations:** 1 Department of Experimental and Clinical Pharmacology, Medical University of Warsaw, Banacha 1B St., 02-097 Warsaw, Poland; 2 2nd Department of Neurology, Institute of Psychiatry and Neurology, Jana III Sobieskiego 9 St, 02-957 Warsaw, Poland; 3 Department of Medical Psychology and Psychotherapy, Erasmus MC, Erasmus University, PO Box 2040, 3000 CA Rotterdam, The Netherlands; 4 Department of Pharmacy Systems, Outcomes, and Policy, College of Pharmacy, University of Illinois at Chicago, 833 South Wood St, Chicago, IL MC886 USA

**Keywords:** EQ-5D-5L, EQ-5D-3L, Health-related quality of life, Patient-reported outcomes, Psychometrics, Stroke

## Abstract

**Aims:**

To date, evidence to support the construct validity of the EQ-5D-5L has primarily focused on cross-sectional data. The aims of this study were to examine the responsiveness of EQ-5D-5L in patients with stroke and to compare it with responsiveness of EQ-5D-3L and visual analogue scale (EQ VAS).

**Methods:**

We performed an observational longitudinal cohort study of patients with stroke. At 1 week and 4 months post-stroke, patients were assessed with modified Rankin Scale (mRS) and Barthel Index (BI) and were administered the EQ-5D-5L and EQ-5D-3L, including the EQ VAS. The EQ-5D-5L index scores were derived using the crosswalk methodology developed by the EuroQol Group. We classified patients according to two external criteria, based on mRS or BI, into 3 categories: ‘improvement,’ ‘stable’ or ‘deterioration’. We assessed the responsiveness of each measure in each patient subgroup using: effect size (ES), standardized response mean (SRM), *F*-statistic, relative efficiency and area under the receiver operating characteristic curve.

**Results:**

A total of 112 patients (52 % females; mean age 70.6 years; 93 % ischemic stroke) completed all the instruments at both occasions. In subjects with clinical improvement, EQ-5D-5L was consistently responsive, showing moderate ES (0.51–0.71) and moderate to large SRM (0.69–0.86). In general, EQ-5D-3L index appeared to be more responsive (ES 0.63–0.82; SRM 0.77–1.06) and EQ VAS less responsive (ES 0.51–0.65; SRM 0.59–0.69) than EQ-5D-5L index.

**Conclusions:**

The EQ-5D-5L index, based on the crosswalk value set, seems to be appropriately responsive in patients with stroke, 4 months after disease onset. As far as EQ-5D-5L index is scored according to crosswalk approach, the EQ-5D-3L index appears to be more responsive in stroke population.

## Introduction

The EQ-5D is a standardized preference-based measure of health that provides a simple, generic measure for clinical and economic assessment [1, 2]. Its classical, three-level version (now called EQ-5D-3L) is successfully used as a secondary outcome in contemporary stroke trials [3]. A 5-level version of the EQ-5D (EQ-5D-5L) was developed with the goal of improving the sensitivity and other psychometric properties of the original EQ-5D-3L [4, 5].

Janssen and colleagues, in a cross-sectional multi-country study, reported evidence of the feasibility and validity of the EQ-5D-5L in a variety of conditions, showing a low level of missing values, establishing known-groups validity and showing improved discriminatory power and improved convergent validity in comparison with EQ-5D-3L [6]. In the context of two studied populations with stroke (from UK and from Poland), a 15 % relative reduction in the ceiling was shown, as well as a valid redistribution and the highest number of different health states defined by the questionnaire, in comparison with other patients groups. Additional studies have been conducted in Germany, Italy, China, South Korea and Singapore that also support the validity of the EQ-5D-5L [7–11]. However, there is a scarcity of studies that have examined the longitudinal construct validity (i.e., responsiveness to change) of the EQ-5D-5L.

Responsiveness is the ability of an outcome instrument to detect clinically important changes within individuals with a specific condition [12]. It is evaluated in longitudinal studies of patients, in whom a change is expected to occur. In general, disease-specific instruments are more responsive than generic health status measures, as they are more focused on problems of particular importance to target patients. Although we have some knowledge about cross-sectional validity of EQ-5D-5L in stroke patients [6], we know very little about its responsiveness in stroke population.

Our objective was to evaluate the responsiveness of the EQ-5D-5L in a longitudinal study of patients with stroke. The secondary objective was to compare responsiveness of EQ-5D-5L index and other generic measures of health-related quality of life (HRQoL), often used in patients with stroke—EQ-5D-3L and EQ VAS.

## Methods

### Study design

A single-center observational longitudinal cohort study was conducted between July 2009 and May 2010. Three neurologists with experience in the use of clinical measures assessed patients with primary or recurrent stroke, at two occasions. Adult patients with primary intracerebral hemorrhage or cerebral infarction (I61 or I63 according to ICD-10 classification) were included. A diagnosis had to be supported by clinical examination and computed tomography (CT) or magnetic resonance imaging (MRI). Individuals had to be Polish language native speakers. Patients in coma were excluded. In case of aphasia or dementia, the survey was administered to family members serving as a proxy.

The initial survey took place during index hospitalization, before discharge. Stroke severity was assessed with the National Institute of Health Stroke Scale (NIHSS) and the modified Rankin Scale (mRS), physical performance with the Barthel Index (BI), and HRQoL with the EQ-5D generic questionnaire (both five- and three-level versions) and the EQ-5D visual analogue scale (EQ VAS). Stroke type was classified according to Oxford Community Stroke Project (OCSP) classification into: partial anterior circulation stroke (PACS), posterior circulation stroke (POCS), lacunar stroke (LACS) or total anterior circulation stroke (TACS). The second survey was conducted after an initial post-stroke recovery phase about 4 months later, in outpatient clinics, neurological rehabilitation department or patients own home. Assessment were completed for the mRS, BI, EQ-5D-5L, EQ-5D-3L and EQ VAS using paper and pencil versions of the quality of life questionnaires.

The study conformed to the Helsinki declaration. The study protocol was approved by the local Ethics Committee, and all participants gave informed consent before inclusion.

### Measures

The mRS and the BI are widely used stroke outcome measures in clinical trials and everyday practice [3]. The mRS is a standardized scale, with good intra-observer agreement, that evaluates the degree of disability or dependence in the daily activities of people who have suffered a stroke or neurological disability, providing a score that ranges from 0 (perfect health without symptoms) to 6 (death) [13]. BI is a valid measure of activities of daily living with a substantial body of literature describing its clinimetrics [14]. It has good reliability and reasonable responsiveness. Although sensitivity to change is limited at the extremes of disability (floor and ceiling effects), BI seems to be more sensitive than other common stroke scales [15]. We used the 10-item scale, scoring 0–100 with 5-point increments [16].

EQ-5D is a brief measure of health that has been used extensively in stroke [17]. It is available for self-completion or by proxy using paper and pencil or electronic versions (PDA, tablet and WWW). Both the EQ-5D-3L and EQ-5D-5L consist of 2 parts: a descriptive health classifier system and a visual analogue scale (EQ VAS). The EQ-5D-5L descriptive system comprises the same five dimensions as the EQ-5D-3L (mobility, self-care, usual activities, pain/discomfort and anxiety/depression), but has five levels of severity (no problems, slight problems, moderate problems, severe problems and extreme problems) rather than three levels (no problems, some problems and extreme problems) in EQ-5D-3L. The responses for the five dimensions can be combined in a 5-digit number describing the respondent’s health state (from ‘11111’ meaning no problems at all to ‘55555’ meaning extreme problems in all five dimensions) [5]. A total of 243 and 3,125 possible health states are defined in this way in EQ-5D-3L and EQ-5D-5L, respectively. EQ-5D health states may be converted into a single summary index by applying a formula that attaches values (also called weights) to each of the levels in each dimension that can facilitate cost–utility analyses. To obtain EQ-5D-3L index values, we used the Polish EQ-5D-3L value set derived using the time trade-off valuation technique [18] and to obtain EQ-5D-5L index scores, we used Polish interim EQ-5D-5L value set estimated using the crosswalk methodology developed by the EuroQol Group [19, 20]. The EQ VAS derives information about the respondents’ subjective health perception, scored on a 20-cm visual analogue scale with endpoints labeled ‘the best health you can imagine’ and ‘the worst health you can imagine.’

### Responsiveness

Responsiveness has been defined as the ability to detect changes that are meaningful or clinically important [21]. To assess responsiveness, some criterion is needed to identify whether patients have changed (either improved or worsened) over time [22]. It is strongly recommended to use multiple independent anchors [23]. We used two external criteria (EC) based on clinical outcomes, namely mRS and BI change scores. The first criterion was based on movement between categories on the mRS at baseline and follow-up: improvement of at least one level (improvement), no movement (stable) and decline at least one level (deterioration). We made no differentiation between ‘some’ change and ‘large’ change, as there were only a small subset of patients who changed two or more levels. There are several different ways with different cut points to categorize BI outcomes [24]. Due to lack of consensus on the approach, we used the minimal clinically important difference (MCID) of the BI in stroke patients estimated by Hsiech et al. [25] of 1.85 points on a 20-point scale (or 9.25 on a 100-point scale).The BI criterion was defined as follows: improvement of at least 9.25 points (improvement), deterioration of at least 9.25 points (deterioration), deterioration of <9.25 points, no change or improvement of <9.25 points (stable).

### Analysis

First, correlations between the change scores of the measures were examined using Spearman’s rank correlation coefficient (*r*_s_). The extent of correlation was interpreted as absent (<0.20), poor (0.20–0.34), moderate (0.35 - 0.50) or strong (>0.50) [26].

Responsiveness was evaluated using the following statistical approaches: (1) effect size, (2) standardized response mean, (3) *F*-statistic, (4) relative efficiency and (5) area under the receiver operating characteristic curve. We calculated effect size (ES) as the ratio of the mean change to the standard deviation (SD) of initial measurement, standardized response mean (SRM) as the ratio of the mean change to the SD of that change and the *F*-statistic as a squared *t*-statistic (squared ratio of the mean change to the standard error of that change). The ES construct ignores the variation in the change, and the SRM construct makes it less sensitive to sample sizes than ES [27]. Both ES and SRM were interpreted as large (>0.8), moderate (0.5–0.8) or small (<0.5) [28, 29]. In analyzing test statistics, a measure that generates the largest statistic is judged to be the most responsive. To compare the responsiveness of measures, relative efficiency (RE) was calculated by taking a ratio of *F*-statistics, where the measure with the smallest *F*-statistic served as the reference, which results in all coefficients being greater than 1.

Receiver operating characteristic (ROC) curves were used to assess the sensitivity and specificity of different change scores [30]. We calculated the size of the area under the curve (AUROC), which corresponds to the probability of correctly identifying patients with a specified outcome according to the EC. AUROC may range from 0.5 (no discriminatory accuracy) to 1.0 (perfect accuracy) in distinguishing patients identified by this criterion [27]. We performed three groups of comparisons: improved versus stable, deteriorated versus stable and improved versus deteriorated patients.

The statistical software used was the StatsDirect 2.7.8 (StatsDirect Ltd, England). The area under the ROC was estimated by a nonparametric method analogous to the Wilcoxon/Mann–Whitney test [31]. Accompanying confidence intervals were constructed using DeLong’s variance estimate [32]. All tests were two-sided. The results were considered significant at *P* < 0.05.

## Results

One hundred and fourteen patients were followed up for about 4 months (median 107.5 days; interquartile range (IQR) 101–123) after the initial stroke hospitalization and after a median of 98.5 days (IQR 93–111) since the first survey. Two patients were excluded from the final analysis, because of missing data: one on initial and follow-up mRS and one on follow-up EQ-5D-5L and EQ-5D-3L. Characteristics of the included 112 subjects are presented in Table 1. The majority of patients had secondary (32 %) or higher (22 %) education, were retired (71 %) or pensioners (15 %), lived with their relatives (79 %) or lived independently (20 %). Comorbidities were common in the studied population: hypertension in 72 % of patients, coronary artery disease in 31 %, diabetes in 25 %, atrial fibrillation in 21 % and cardiac insufficiency in 16 %. Thirty-one percent of subjects were current smokers. The most common stroke symptoms included: upper extremity (79 %) or lower extremity paresis (64 %), dysphasia (33 %), hemianopsia (16 %), dysarthria (31 %) or brain stem or cerebral sings (15 %). In 54 % and 40 % of patients, stroke involved right and left side of the body, respectively, with no obvious side affected in 4 %. According to OCSP stroke classification, the sample was composed of: 46 % PACS, 26 % POCS, 20 % LACS and 7 % TACS. For 20 % of patients, it was recurrent stroke. Median hospital stay was 10 days (IQR 8–14 days), and median intensive care unit stay was 1 day (IQR 0–2 days). Patients were discharged to their own house (77 %), rehabilitation ward (17 %) or transferred to another hospital (5 %).

**Table 1:** Demographic characteristics of studied population with stroke

	First survey	Follow-up
*N*	112	
Age, years		
Mean (SD)	70.6 (11.0)	
Range	39–88	
Sex, F, *n* (%)	58 (51.8)	
ICD-10, *n* (%)		
I61 (intracerebral hemorrhage)	8 (7.1)	
I63 (cerebral infarction)	104 (92.9)	
mRS, *n* (%)		
0	2 (1.8)	5 (4.5)
1	23 (20.5)	32 (28.6)
2	42 (37.5)	42 (37.5)
3	21 (18.8)	16 (14.3)
4	14 (12.5)	8 (7.1)
5	10 (8.9)	9 (8.0)
NIHSS		
Mean (SD)	4.1 (4.8)	
Assessment site, *n* (%)		
Hospital ward	105 (93.8)	4 (3.6)
Outpatient clinic	5 (4.5)	85 (75.9)
Rehabilitation ward	2 (1.8)	3 (2.7)
Home	0 (0)	20 (17.9)
Respondent, *n* (%)		
Patient	91 (81.3)	102 (91.1)
Proxy	21 (18.7)	10 (8.9)

*mRS* modified Rankin scale, *NIHSS* National institute of health stroke scale

Between baseline and follow-up, all clinical and HRQoL measures showed improvement based on mean and median scores (paired *t* tests all <0.01; Table 2). Significant differences in the distribution of responses to self-care and usual activities EQ-5D-5L dimensions were observed (Chi-squared tests <0.001 and 0.001, respectively) (Table 3).

**Table 2:** Descriptive statistics for HRQoL and clinical outcome measures

	Mean (SD)	Median (Q1–Q3)	Range	% Floor	% Ceiling	% Negative
Baseline						
EQ-5D-5L index	0.577 (0.343)	0.724 (0.478–0.791)	−0.523 to 1.0	1.8	5.4	8.0
EQ-5D-3L index	0.584 (0.353)	0.716 (0.369–0.798)	−0.523 to 1.0	2.7	6.3	7.1
EQ VAS	54.3 (24.8)	50 (40–70)	0–100	1.8	3.6	–
Barthel index	78.9 (30.4)	95 (70–100)	0–100	2.7	49.1	–
mRS	2.5 (1.3)	2 (2–3)	5–0	8.9	1.8	–
Follow-up						
EQ-5D-5L index	0.691 (0.267)	0.741 (0.619–0.861)	−0.231 to 1.0	0.0	7.1	4.5
EQ-5D-3L index	0.694 (0.281)	0.768 (0.716–0.868)	−0.523 to 1.0	0.9	9.8	1.8
EQ VAS	60.7 (22.4)	60 (45.5–80)	0–100	0.9	1.8	–
Barthel Index	84.6 (26.3)	100 (80–100)	0–100	3.6	55.4	–
mRS	2.2 (1.3)	2 (1–3)	5–0	8.0	4.5	–

*EQ VAS* EQ-5D visual analogue scale, *mRS* modified Rankin scale

**Table 3:** Distribution of EQ-5D-5L dimension responses at baseline and at follow-up (*N* = 112)

Dimension	Baseline *n* (%)	Follow-up *n* (%)	*P* value*
Mobility			
No problems	17 (15.2)	34 (30.4)	0.057
Slight problems	28 (25.0)	24 (21.4)	
Moderate problems	31 (27.7)	29 (25.9)	
Severe problems	18 (16.1)	16 (14.3)	
Unable to walk about	18 (16.1)	9 (8.0)	
Self-care			
No problems	28 (25.0)	55 (49.1)	<0.001
Slight problems	27 (24.1)	19 (17.0)	
Moderate problems	22 (19.6)	18 (16.1)	
Severe problems	10 (8.9)	12 (10.7)	
Unable to wash or dress	25 (22.3)	8 (7.1)	
Usual activities			
No problems	16 (14.3)	30 (26.8)	0.001
Slight problems	29 (25.9)	27 (24.1)	
Moderate problems	28 (25.0)	26 (23.2)	
Severe problems	10 (8.9)	20 (17.9)	
Unable to do usual activities	29 (25.9)	9 (8.0)	
Pain/discomfort			
No pain or discomfort	24 (21.4)	29 (25.9)	NS
Slight pain or discomfort	26 (23.2)	24 (21.4)	
Moderate pain or discomfort	41 (36.6)	40 (35.7)	
Severe pain or discomfort	19 (17.0)	15 (13.4)	
Extreme pain or discomfort	2 (1.8)	4 (3.6)	
Anxiety/depression			
Not anxious or depressed	20 (17.9)	26 (23.2)	NS
Slightly anxious or depressed	36 (32.1)	44 (39.3)	
Moderately anxious or depressed	33 (29.5)	31 (27.7)	
Severely anxious or depressed	20 (17.9)	9 (8.0)	
Extremely anxious or depressed	3 (2.7)	2 (1.8)	

* Chi-square test, *NS* non significant

Correlation between change scores of each measure revealed that changes in EQ-5D-5L were strongly correlated with EQ-5D-3L, moderately with EQ VAS and BI and poorly with the mRS (Table 4). EQ-5D-3L tended to have stronger levels of correlation with stroke clinical outcome measures than EQ-5D-5L. The weakest observed correlation was between EQ VAS and mRS or BI change scores. Surprisingly, the correlation between mRS and BI change scores was only moderate.

**Table 4:** Correlations between change scores of studied measures (Spearman’s rank correlation coefficient)

	EQ-5D-5L index	EQ-5D-3L index	EQ VAS	Barthel Index	mRS
EQ-5D-5L index	1.00				
EQ-5D-3L index	0.74	1.00			
EQ VAS	0.48	0.41	1.00		
Barthel index	0.43	0.56	0.27	1.00	
mRS	−0.31	−0.41	−0.32	−0.42	1.00

*EQ VAS* EQ-5D visual analogue scale, *mRS* modified Rankin scale

According to our predefined mRS external anchor, slightly more patients were defined as improved (38.4 %) or deteriorated (17.0 %) compared with findings based on the BI as an external anchor (33.0 % and 13.4 %, respectively). Table 5 shows change scores for each measure stratified by subgroup when defined by each of the external criteria (mRS and BI). In general, mean EQ-5D-3L index changes were greater than mean EQ-5D-5L index changes, and the latter were greater than EQ VAS changes.

**Table 5:** Descriptive statistics (mean, SD) for HRQoL and clinical outcome measures in patients classified as improved, stable or deteriorated, according to external criterion

Measure	Time point	mRS-based external criterion			Barthel index-based external criterion		
		Improved *N* = 43	Stable *N* = 50	Deteriorated *N* = 19	Improved *N* = 37	Stable *N* = 60	Deteriorated *N* = 15
EQ-5D-5L index	Baseline	0.529 (0.388)	0.590 (0.333)	0.652 (0.246)	0.341 (0.376)	0.716 (0.242)	0.603 (0.304)
	Follow-up	0.729 (0.217)	0.696 (0.292)	0.590 (0.292)	0.607 (0.232)	0.795 (0.177)	0.482 (0.429)
	Change	0.200 (0.290)	0.106 (0.214)	−0.061 (0.247)	0.267 (0.311)	0.078 (0.165)	−0.121 (0.256)
EQ-5D-3L index	Baseline	0.531 (0.382)	0.595 (0.357)	0.674 (0.253)	0.323 (0.377)	0.731 (0.248)	0.637 (0.293)
	Follow-up	0.769 (0.174)	0.691 (0.286)	0.530 (0.150)	0.634 (0.228)	0.796 (0.198)	0.434 (0.445)
	Change	0.239 (0.309)	0.096 (0.189)	−0.144 (0.381)	0.310 (0.294)	0.065 (0.202)	−0.203 (0.352)
EQ VAS	Baseline	51.3 (25.1)	56.9 (25.6)	53.9 (22.7)	38.1 (20.3)	64.5 (22.0)	53.0 (25.4)
	Follow-up	64.1 (19.8)	64.2 (22.0)	43.6 (22.1)	51.3 (17.9)	69.2 (19.3)	49.6 (29.9)
	Change	12.8 (21.5)	7.3 (20.5)	−10.3 (17.4)	13.2 (19.1)	4.72 (24.1)	−3.4 (11.2)

*EQ VAS* EQ-5D visual analogue scale, *mRS* modified Rankin scale

In the analysis based on external criteria, both ES and SRM are higher when patients were classified as ‘improved,’ rather than ‘deteriorated.’ In subjects who improved, indices showed at least moderate responsiveness, with responsiveness statistics associated with the EQ-5D-3L index being consistently more responsive than EQ-5D-5L index. In patients who improved based on the BI, both the EQ-5D-3L index and the EQ-5D-5L captured large magnitudes of effect according to the SRM. A similar pattern was observed using mRS as the basis for categorizing patients into outcome groups (Table 6).

**Table 6:** Responsiveness statistics for HRQoL and clinical measures by external criterion

Responsiveness statistic	Measure	mRS-based external criterion		Barthel index-based external criterion	
		Improved *N* = 43	Deteriorated *N* = 19	Improved *N* = 37	Deteriorated *N* = 15
Effect size	EQ-5D-5L index	0.51	−0.25	0.71	−0.40
	EQ-5D-3L index	0.63	−0.57	0.82	−0.69
	EQ VAS	0.51	−0.45	0.65	−0.13
Standardized response mean	EQ-5D-5L index	0.69	−0.25	0.86	−0.47
	EQ-5D-3L index	0.77	−0.38	1.06	−0.58
	EQ VAS	0.59	−0.59	0.69	−0.30
*F*-statistic	EQ-5D-5L index	20.32	1.17	27.15	3.36
	EQ-5D-3L index	25.68	2.70	41.30	4.98
	EQ VAS	15.25	6.72	17.65	1.38
Relative efficiency	EQ-5D-5L index	1.33	1.00	1.54	2.43
	EQ-5D-3L index	1.68	2.31	2.34	3.60
	EQ VAS	1.00	5.75	1.00	1.00

*EQ VAS* EQ-5D visual analogue scale, *mRS* modified Rankin scale

Responsiveness analysis based on ROC curves similarly found that the BI worked better than mRS as an external criterion, giving higher AUROC, which indicates better accuracy. Systematically, the most responsive measure was the EQ-5D-3L index. When the external criterion was based on BI, the second most responsive instrument was the EQ-5D-5L, but when EC was based on mRS, the second was EQ VAS (Table 7).

**Table 7:** Area under the receiver operating characteristic curve (AUROC) analysis

Compared populations	Measure	AUROC (95 % CI)	
		External criterion: mRS	External criterion: BI
Improved versus stable	EQ-5D-5L index	0.57 (0.45–0.69)	0.71 (0.59–0.83)
	EQ-5D-3L index	0.63 (0.52–0.75)	0.79 (0.69–0.89)
	EQ VAS	0.58 (0.46–0.69)	0.62 (0.50–0.73)
Deteriorated versus stable	EQ-5D-5L index	0.70 (0.44–0.95)	0.70 (0.41–1.00)
	EQ-5D-3L index	0.74 (0.45–1.00)	0.75 (0.43–1.00)
	EQ VAS	0.74 (0.45–1.00)	0.63 (0.43–0.82)
Improved versus deteriorated	EQ-5D-5L index	0.75 (0.62–0.89)	0.83 (0.72–0.94)
	EQ-5D-3L index	0.81 (0.69–0.93)	0.91 (0.84–0.98)
	EQ VAS	0.80 (0.69–0.92)	0.75 (0.62–0.88)

*AUROC* area under the receiver operating characteristic curve, *BI* Barhel index, *mRS* modified Rankin scale

## Discussion

In the present study, the EQ-5D-5L showed appropriate responsiveness in patients about 4 months after stroke, as confirmed by using several indices—ES, SRM, *F-*statistic, RE and area under the ROC curve. We noticed moderate ESs and moderate to large SRMs. Nevertheless, in our sample of stroke patients, EQ-5D-3L index appeared to be more responsive than EQ-5D-5L index scored according to crosswalk approach. Contrary, EQ VAS showed to function worse in the studied context.

The finding that in stroke patients EQ-5D-5L appears to be less responsive than EQ-5D-3L may be seen unexpected, as five-level version was developed with the goal of improving psychometric properties of the three-level EQ-5D. It should be viewed in terms of limitations of the value set we used. An important limitation of the current study was reliance on an interim EQ-5D-5L value set derived from a crosswalk algorithm [20]. Index scores based on mapping functions are less reliable than scores from value sets based on preferences directly elicited from representative general population samples. In other study, we found that the Polish interim EQ-5D-5L value set generated values to some extent more narrow, than those generated by the EQ-5D-3L time trade-off value set. There were relatively less health states valued ‘worse than death’ or as a good health and, at the same time, relatively more health states valued moderately [20]. Moving from ‘bad health’ to ‘good health’ resulted in a smaller change in the EQ-5D-5L index value based on crosswalk methodology than in the directly measured EQ-5D-3L index value. Although many approaches were explored by the EuroQol research team that published the crosswalk algorithm [19], it should be considered second best to direct utility measurement. At the moment, directly measured value sets for EQ-5D-5L are under development [33, 34]. EQ-5D-5L responsiveness properties should be revisited when these sets become available.

There is no ‘gold standard,’ i.e., which measure is superior in establishing whether a relevant or significant change in HRQoL of stroke patients has occurred. The use of multiple clinical anchor-based criteria of change is advised. In the present study, we identified patients with improvement or worsening based on a general disability measure—mRS and a physical performance based measure—BI. It can be argued that the use of a different HRQoL measure as an anchor may give more reliable results, but both mRS and BI are recognized as the most often used standard of stroke outcome measurement [3] and were successfully used in studies of responsiveness of preference-based generic HRQoL measures in stroke [35].

Responsiveness of studied instruments was higher when external criterion was based on BI, rather than mRS. It can be that the studied indices are closely correlated with BI or that our mRS measurement had limited reliability. The mRS is the preferred measure of disability in stroke trials, but its value is restricted by potentially significant interobserver variability [36, 37]. Several attempts were made to reduce the bias between mRS raters, such as introduction of a structured interview, video-based training and certification, but effects have not been consistent [38]. Another explanation could be that we allowed the use of proxies, in case of aphasia or dementia. In our study, surveys performed in this way constituted less than 20 % during the first survey and less than 10 % at follow-up. Some authors reported that patient’s assessment of HRQoL has a stronger association with mRS, while proxy responses have a stronger association with BI [39].

Contrary to other authors, we made no differentiation between some and large improvement, as there were only some patients with a shift of two or more levels in mRS [35]. We also did not exclude patients who deteriorated. Final numbers of patients with deterioration, according to mRS and BI external anchors, were low (19 and 15, respectively), posing a question about validity and generalizability of results obtained in these groups.

To our best knowledge, this is the first study of EQ-5D-5L responsiveness in patients with stroke. The three-level version of EQ-5D was investigated in this context, twice. Hunger and colleagues showed reasonable validity, reliability and more limited responsiveness of a sample of German patients with a history of stroke, mild to moderate limitations of functional status, undergoing neurological rehabilitation [17]. Observed ESs were lower than estimated by Pickard et al. [35] and in our study. A possible explanation is that patients in the German study were included later after the stroke onset (median 5.7 weeks) and characterized with better functional status. As noticed by Pickard et al., the EQ-5D index is highly responsive in conditions where extreme health problems are encountered initially and subsequently improve. EQ-5D, with five dimensions and three levels, was as efficient in capturing changes as SF-6D, with six dimensions and four to six levels and Health Utility Index-3 (HUI3), with eight dimensions and five or six levels [35].

Our study is one of the first to examine the responsiveness of the EQ-5D-5L. Up till now, this topic was investigated only in women with breast cancer [11] and patients undergoing colonoscopy [40]. Swan and colleagues stated that EQ-5D-5L is unresponsive in colonoscopy patients, with the SRM and the ES moderately positive and a significant baseline to post-procedure change in the direction unexpected by authors. In contrast, Lee and coauthors, assessing EQ-5D-5L responsiveness in breast cancer patients, found it reasonably responsive with ES = 0.52 and 0.69, when external criterion was based on self-assessed performance status or self-rated change in quality of life, respectively.

Future studies of the EQ-5D-5L in stroke patients should, also, provide an in-depth look at its validity and reliability, especially in the context of test–retest reliability.

We conclude that the EQ-5D-5L index, based on the crosswalk value set, seems to be appropriately responsive in patients with stroke, 4 months after disease onset. As far as EQ-5D-5L index is scored according to crosswalk approach, the EQ-5D-3L index appears to be more responsive in stroke population.
